# Microbiota-gut-brain axis pathogenesis and targeted therapeutics in sleep disorders

**DOI:** 10.3389/fneur.2025.1721606

**Published:** 2025-12-12

**Authors:** Dan Pan, Jinyi Li, Siyu Chen, Simeng Gu, Mingchen Jiang, Qiuyue Xu

**Affiliations:** 1 School of Nursing, Nanjing University of Chinese Medicine, Nanjing, China; 2 Department of Pediatrics, Affiliated Hospital of Nanjing University of Chinese Medicine, Nanjing, China; 3 Department of Psychology, Jiangsu University Medical School, Zhenjiang, China

**Keywords:** sleep disorders, microbiota-gut-brain axis, gut microbiota, microbial metabolites, targeted therapeutics

## Abstract

Sleep constitutes an essential physiological process that is vital for maintaining physical and mental wellbeing. However, the science of sleep focusing on basic questions such as “how” we sleep and “why” we sleep is still not clear. Over the past decade, substantial progress has also been made in elucidating the interactions between sleep and other biological processes, providing insights into the basic questions of sleep. Among these, emerging evidence highlights the microbiota-gut-brain axis (MGBA) as a pivotal bidirectional network that connects gut microorganisms with the central nervous system to regulate sleep architecture and homeostasis. This interaction is inherently bidirectional: sleep deprivation alters gut motility, mucosal integrity, and microbial composition, while microbial metabolites in turn influence neurotransmission (*γ*-aminobutyric acid, serotonin), immune-endocrine balance, and inflammatory signaling. In this article, we will review recent studies about MGBA-targeted therapeutic strategies for sleep disorders, such as probiotics, prebiotics, and fecal microbiota transplantation, which aim to restore microbial homeostasis and improve sleep quality. Furthermore, we discuss emerging interventions that modulate microbial metabolites and neuroimmune-endocrine signaling, as well as innovative pharmacological approaches targeting MGBA dysfunction. Collectively, we hope this review will contribute to a deeper understanding of MGBA-mediated mechanisms in sleep disorders promises to inform novel preventive and therapeutic strategies, ultimately improving clinical outcomes and quality of life for affected individuals.

## 1 Introduction

Sleep constitutes an essential and intricate physiological process that is fundamental to the maintenance of both physical and mental wellbeing. Over the past decade, our understanding of the sleep-control mechanism has advanced rapidly, and substantial progress has also been made in elucidating the interactions between sleep and other biological processes (1, 2). Insomnia is remarkably common in clinical settings, affecting nearly half of all primary care patients (1). It can manifest either in isolation or as a comorbidity with other medical and psychiatric disorders; if left untreated, insomnia may exacerbate the risk of developing or worsening these concomitant conditions (3, 4). In the elderly population, insomnia is frequently associated with comorbidities such as dementia, depression, anxiety disorders, and chronic pain, further complicating its clinical management (5).

Early research on sleep mechanisms primarily focused on the central nervous system (CNS) regulation of sleep–wake cycles (6). The hypothalamic–pituitary–adrenal (HPA) axis, a core regulatory pathway, precisely modulates sleep architecture: morning cortisol peaks facilitate awakening, whereas nocturnal declines support deep sleep. Cortisol follows a 24-h circadian rhythm, playing a critical role in aligning physiological systems with behavioral cycles (7, 8). Notably, sleep disturbances and stress exposure interact bidirectionally. Chronic stress can hyperactivate the HPA axis, elevating nocturnal cortisol levels and thereby impairing sleep initiation and depth. Conversely, recurrent sleep disruption diminishes HPA axis regulatory capacity, increasing vulnerability to stress and establishing a persistent dynamic feedback loop between stress and sleep quality (9, 10). However, recent studies found that central regulatory mechanisms alone cannot fully account for the complexity of sleep homeostasis. For instance, why recovery sleep following deprivation preferentially enhances deep sleep rather than merely extending total sleep time (11), and why sleep disturbances are strongly associated with systemic conditions such as cardiovascular and metabolic diseases (12) and cognitive decline (13). These observations suggest that sleep regulation is not solely a “central” process, but rather requires the coordinated involvement of peripheral organs. Against this backdrop, increasing evidence indicates that peripheral systems, particularly the gut, play a critical role in modulating sleep physiology. Within the research framework examining central-peripheral interactions, the microbiota-gut-brain axis (MGBA) has emerged as a key mechanistic bridge linking these two domains.

**Figure 1:**
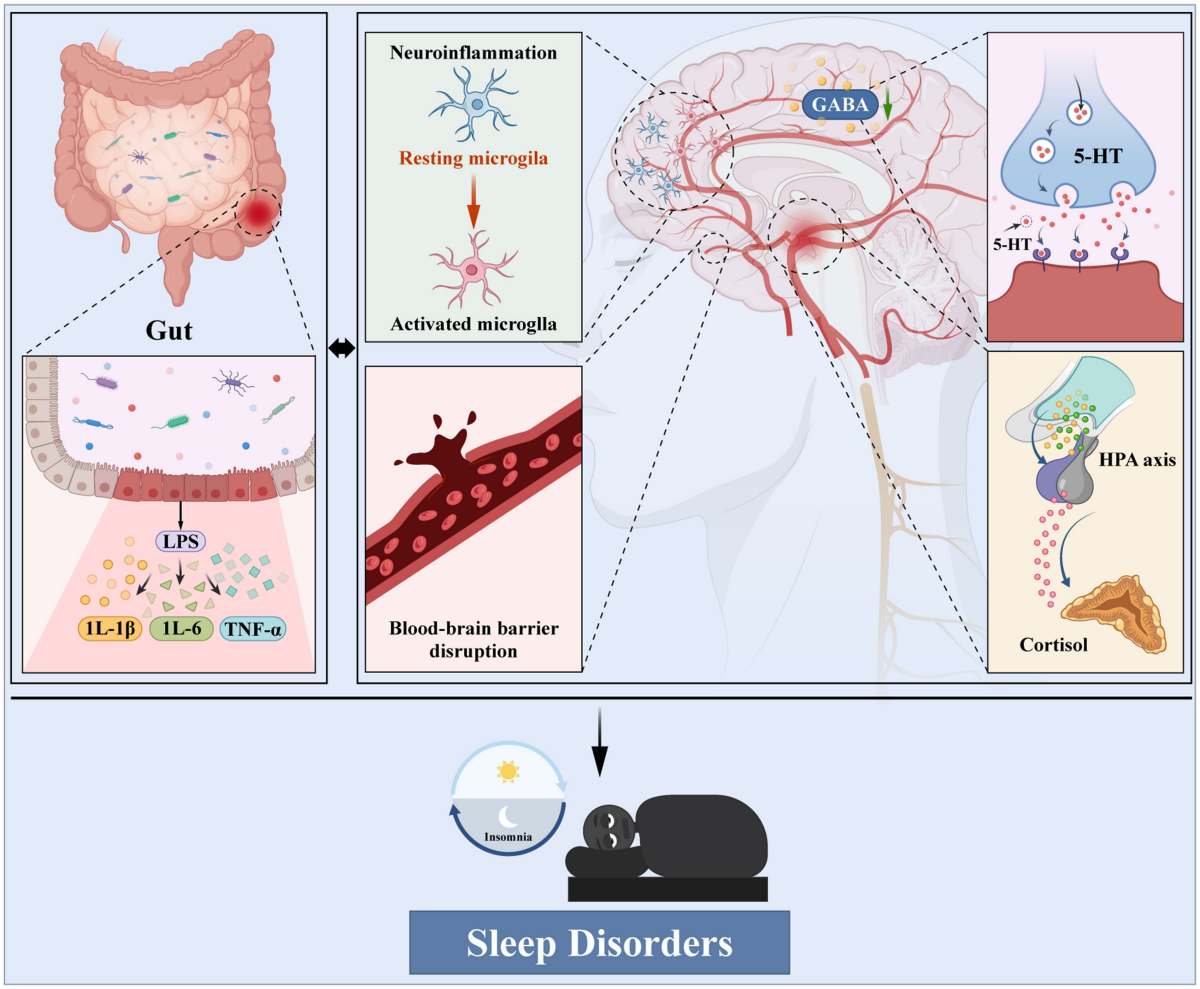
Intestinal barrier damage, flora disorder and microglia activation, destroy the blood–brain barrier, affect the function of neurotransmitters, and drive the HPA axis and multi-system linkage eventually leads to sleep disorders.

Gut microbes can synthesize or modulate neurotransmitters such as *γ*-aminobutyric acid (GABA), serotonin (5-HT), and dopamine, directly influencing neural circuits that govern sleep–wake balance (14). In addition, gut-derived inflammatory signals-such as lipopolysaccharide (LPS) and pro-inflammatory cytokines including interleukin-6 (IL-6), tumor necrosis factor-*α* (TNF-α), and interleukin-1β (IL-1β) can disrupt blood–brain barrier (BBB) integrity, activate microglia, and provoke neuroinflammation, contributing to sleep disturbances (15). The HPA axis, in turn, links sleep stress with microbial dysbiosis: elevating cortisol levels, enhancing gut permeability and reducing mucin production, weakening intestinal barrier integrity and amplifying immune activation. These feedback loops underscore the bidirectional and self-reinforcing cycle between the gut and the brain in sleep disorders (16, 17). Additionally, sleep itself modulates gut physiology. Experimental models demonstrate that chronic sleep restriction disrupts tight junction proteins (occludin, claudin-1), reduces mucosal mucus secretion, alters enteric nervous system excitability, and accelerates intestinal permeability. These effects compromise microbial habitat stability, leading to dysbiosis that perpetuates inflammatory signaling and sleep fragmentation. Together, these pathways illustrate the integrative and bidirectional role of the MGBA in sleep regulation (Figure 1).

Aging further modulates the MGBA-sleep interplay, rendering older adults a high-risk population for sleep disorders. Epidemiological data indicate that the prevalence of insomnia is significantly higher in older adults compared with younger populations, with approximately 41–48% of individuals over 60 years experiencing difficulties initiating sleep, shallow sleep, or sleep fragmentation (18, 19). These phenomena are closely linked to age-related dysregulation of MGBA function. At the level of gut microbiota (GM), characteristic alterations occur in the elderly: on one hand, the abundance of potentially beneficial bacteria, such as *faecalibacterium prausnitzii*, decreases, while the abundance of pathogenic bacteria increases (20); on the other hand, low-grade inflammation driven by increased pro-inflammatory bacteria can transmit inflammatory mediators to the CNS via the bloodstream, leading to difficulties in sleep initiation and reduced sleep depth (21, 22). Conversely, impaired sleep quality in older adults further exacerbates GM dysbiosis, creating a vicious cycle (23).

This review aims to synthesize current evidence on the MGBA’s role in sleep disorders, from mechanistic foundations to clinical translation. Tracing the evolution of MGBA research helps contextualize its growing relevance in sleep medicine. Epidemiological and clinical evidence increasingly links gut dysbiosis to sleep abnormalities, influenced by stress, and circadian rhythms. A thorough understanding of these foundational aspects is crucial for developing novel diagnostic and therapeutic strategies aimed at modulating the MGBA to improve sleep health.

## 2 Microbiota-gut-brain axis in sleep disorders

Research on the MGBA has increasingly focused on its role in the pathophysiology of sleep disorders. This bidirectional communication network connects the gut with the CNS, with the GM playing a central role in this dialogue, thereby modulating numerous physiological processes, including sleep regulation. Evidence suggests a reciprocal relationship within this communication: alterations in GM may affect sleep architecture, while sleep disruptions can induce alterations in the microbial community structure (24). Washed microbiota transplantation represents a promising therapeutic strategy, demonstrating efficacy in significantly enhancing sleep quality among individuals with sleep disorders. Washed microbiota transplantation not only shortens sleep latency and prolongs sleep duration but also improves overall quality of life by restoring dysregulated GM homeostasis (25). This suggests that microbiota-targeted therapies could be effective in managing sleep disorders by reestablishing microbial equilibrium.

Beyond sleep-specific implications, the MGBA also intersects with broader health conditions such as metabolic syndrome. As a key communication pathway, the MGBA integrates gut microbial signals with central sleep regulation. Recent studies have identified shared pathological biomarkers and mechanistic overlaps between sleep disturbances and metabolic syndrome within this axis (26). Additionally, beyond metabolic interactions, the bidirectional interaction between GM and the nervous system plays a vital role in maintaining homeostasis. Intestinal dysbiosis has been associated with the onset and progression of various neurological disorders, including Alzheimer’s and Parkinson’s diseases. By modulating the production and release of neurotransmitters, the GM significantly affects CNS function, thereby establishing a strong rationale for microbiota-targeted interventions in the management of neurological disorders (27). Emerging evidence implicates the MGBA in the pathogenesis of sleep deprivation-induced cognitive impairment. Specifically, chronic sleep loss induces intestinal dysbiosis and promotes activation of the NOD-like receptor family pyrin domain-containing 3 (NLRP3) inflammasome within both colonic and cerebral tissues, ultimately contributing to cognitive deficits. Targeting the NLRP3 inflammasome within the MGBA could offer therapeutic strategies for cognitive impairment associated with sleep disorders (15).

In conclusion, the MGBA is a critical component in understanding and treating sleep disorders. A deeper investigation into the complex bidirectional interplay between the GM and CNS signaling paves the way for novel therapeutic strategies that target the root etiology of sleep disturbances, thereby enhancing patient wellbeing and long-term health. The following sections will elaborate on the central pathological mechanisms by which dysregulated microbial metabolites, immune-mediated inflammation activation, neuroendocrine disturbances, and aberrant vagal nerve signaling contribute to sleep disorders within the MGBA framework.

## 3 Key pathophysiological mechanisms of the microbiota-gut-brain axis in sleep disorders

The MGBA constitutes a dynamic bidirectional network that connects intestinal microorganisms with the central nervous system. Within this framework, several interrelated mechanisms, such as neurotransmitter regulation, neuroimmune interaction, neuroendocrine control, and microbial metabolite signaling, work together to sustain sleep–wake homeostasis. Disturbance of this delicate equilibrium contributes to the development and maintenance of sleep disorders.

### 3.1 Microbial neuromodulation and neuroendocrine regulation

GM-derived metabolites, including glutamate, GABA, 5-HT, and dopamine, act as key neurotransmitters or neuromodulators (14). Among these, 5-HT is the major neurotransmitter for sleep in the brain, which is released by raphe nucleus. However, 95% of serotonin is release in the gut, and might be recycled to the brain to affect sleep (28). GABA, a major inhibitory neurotransmitter, is synthesized in GABAergic neurons via the decarboxylation of glutamate catalyzed by glutamate decarboxylase. Notably, several gut bacterial species are also capable of producing GABA. Beyond its central roles, GABA also signals within the enteric nervous system via specific receptors, influencing gut neural activity and potentially modulating central arousal (29).

The HPA axis, a central neuroendocrine stress response system, plays a critical role in sleep regulation. Elevated levels of corticotropin-releasing hormone, adrenocorticotropic hormone, and cortisol are associated with increased wakefulness and reduced non-rapid eye movement sleep (16). Sleep impairment initially enhances neuroendocrine stress reactivity, but when sleep disorders and/or stress experiences persist for a longer period, the sensitivity of the HPA axis may eventually decrease. The bidirectional relationship between sleep and the secretion activity of the HPA axis further complicates the complexity of the causal relationship. Therefore, while sleep disturbances are known to increase the secretory activity of the HPA axis, evidence indicates that heightened HPA sensitivity may, in turn, contribute to sleep insufficiency (30). Importantly, gut dysbiosis, which is marked by changes in the Firmicutes-to-Bacteroidetes ratio and an increased abundance of pathobionts, can impair intestinal barrier integrity, allowing LPS translocation and triggering localized immune activation. This includes the activation of macrophages and T cells within the lamina propria, leading to increased production of pro-inflammatory cytokines such as IL-6 and TNF-*α*. Concurrently, reduced levels of microbiota-derived short-chain fatty acids (SCFAs) weaken anti-inflammatory signaling, further elevating IL-6 levels and disrupting circadian rhythmicity, thereby adversely affecting sleep. Additionally, GM-derived signals can prime the NLRP3 inflammasome via the toll-like receptor 4/nuclear factor kappa-B (TLR4/NF-κB) pathway, which promotes the transcription of NLRP3 and pro-IL-1β. Subsequent activation of the inflammasome stimulates TNF-*α* release and contributes to BBB disruption (15). In addition, probiotics modulate HPA axis activity. *Bifidobacterium longum* and *Lactobacillus helveticus* attenuate stress-induced visceral hypersensitivity, while long-term *B. longum infantis* administration mitigates HPA hyperactivity and improves emotional behavior and brain-derived neurotrophic factor signaling (31, 32). These findings illustrate the bidirectional communication between GM and neuroendocrine pathways.

### 3.2 Neuroimmune and inflammatory crosstalk in sleep disorders

The interaction between sleep and immune function is crucial for preserving inflammatory homeostasis and alleviating disease pathogenesis—a process profoundly influenced by the GM. The MGBA serves as a hub for this bidirectional communication. Recent evidence further supports this view: gut-derived neuroimmune signaling has been identified as a key mechanism mediating the bidirectional regulation between intestinal microbiota and sleep physiology, with microbial metabolites and inflammatory mediators jointly shaping sleep homeostasis and neural function (33). Sleep deprivation provokes systemic low-grade inflammation characterized by elevated IL-6, TNF-*α*, and C-reactive protein, while intestinal dysbiosis can exacerbate these inflammatory responses through increased intestinal permeability and microbial translocation (34, 35).

Activated microglia and astrocytes are key mediators linking peripheral inflammation to central neural circuits. IL-1β and TNF-*α* penetrate or signal across the BBB, altering neuronal excitability and impairing slow-wave activity (36). SCFAs and tryptophan derivatives mitigate these effects by suppressing microglial activation and promoting M2 anti-inflammatory polarization, whereas dysbiosis reduces SCFAs availability and promotes neuroinflammation.

Systemic T-cell dynamics, particularly the Th17/Treg balance, further influence CNS immunity and sleep regulation. Experimental studies demonstrate that astragalus polysaccharides can regulate the Th17/Treg balance in experimental autoimmune encephalomyelitis mice by modulating the composition of the GM and influencing gut and plasma metabolite profiles. This immunomodulatory effect correlates with mitigated neuroinflammation and ameliorated experimental autoimmune encephalomyelitis clinical scores, underscoring the therapeutic potential of microbiota-targeted strategies for CNS immune regulation (37). Furthermore, intestinal barrier integrity serves as a first line of defense. Tight junction breakdown and mucin depletion allow LPS and flagellin to trigger TLR4/TLR5-mediated inflammatory signaling. These cytokine cascades not only activate microglia but also alter astrocyte function and neuronal excitability via prostaglandin E2-EP4 signaling (38). Sleep deprivation itself downregulates mucin secretion and goblet cell activity, reinforcing a vicious cycle where impaired barrier function and neuroinflammation perpetuate one another.

### 3.3 Microbial metabolites in sleep regulation

Microbial metabolites, including SCFAs, bile acids, and tryptophan-derived compounds, act as key mediators that connect gut microbial functions with the regulation of sleep in the CNS. SCFAs generated through fermentation of dietary fibers modulate neurotransmission, neuroimmune signaling, and astrocytic metabolism, collectively influencing sleep–wake patterns (39–41). Mechanistically, SCFAs regulate neuronal protein phosphorylation by inhibiting protein kinase A (PKA) and activating protein phosphatases (PPs), thereby maintaining adenosine-mediated excitatory-inhibitory balance (42–44).

Purinergic signaling is similarly regulated: ATP serves as an important modulator of sleep–wake cycles. Adenosine, a key metabolite of ATP breakdown, accumulates during wakefulness and promotes sleep drive by increasing homeostatic sleep pressure (45). Adenosine regulates the neural circuits of the sleep–wake cycle by binding to A1R and A2AR receptors (46). Additionally, ATP itself can directly activate purinergic P2X and P2Y receptors, thereby influencing sleep–wake patterns via both adenosine-dependent and -independent mechanisms (47). SCFAs may further modulate purinergic signaling by affecting ATP availability and receptor expression, adding another layer to the interaction between GM and sleep regulation. These mechanisms collectively underscore the multifaceted roles of ATP and its metabolites in neurotransmitter dynamics and sleep–wake patterning. SCFAs also stabilize slow-wave sleep by maintaining microglial homeostasis, preventing astrocyte neurotoxic transformation, and enhancing astrocytic adenosine release (48, 49).

Antibiotic-induced GM depletion demonstrates causality. Evidence indicates that broad-spectrum antibiotics, while combating infections, frequently induce gut dysbiosis (50), which subsequently leads to significant alterations in sleep architecture (51). This finding strongly supports the essential role of GM in maintaining normal sleep patterns. Mechanistic investigations further reveal that antibiotic-induced dysbiosis disrupts microbial synthesis of neurotransmitters and reduces beneficial metabolite production (52), thereby triggering systemic low-grade inflammation and amplifying corticosterone responses, ultimately impairing restorative sleep. These results clarify key pathways through which antibiotics influence brain function via GM modulation. Notably, despite the widespread clinical use of antibiotics for anti-infective therapy, their application is associated with various potential risks. A Norwegian cross-sectional study demonstrated a positive correlation between short sleep duration, chronic insomnia, and infection incidence as well as frequency of antibiotic use (53). In patients with cystic fibrosis, antibiotic administration has also been linked to an increased risk of sleep apnea syndrome and nocturnal hypoxemia (54). Furthermore, antibiotic exposure is associated with elevated risks of psychiatric conditions such as depression and anxiety, which are themselves significant contributors to sleep disturbances (55). Therefore, clinical anti-infective strategies should incorporate comprehensive assessments of the potential impacts of antibiotics on GM, sleep quality, and mental health (56). Nevertheless, evidence derived from antibiotic interventions remains invaluable for elucidating causal relationships between GM and sleep.

### 3.4 Neuroendocrine clearance and glymphatic function

The glymphatic system serves as a critical waste-clearance network in the brain, with its function peaking during slow-wave sleep (57, 58). In this state, reduced neuronal activity and decreased norepinephrine levels collectively lead to a reduction in brain cell volume and expansion of the extracellular space (59, 60). These structural changes facilitate enhanced cerebrospinal fluid circulation, which effectively clears metabolic by-products such as amyloid-*β*. The functionality of this system is highly dependent on the polarized distribution of aquaporin-4 (AQP4) water channels located on the perivascular endfeet of astrocytes (61). Beyond mediating waste clearance via AQP4, astrocytes play a central role in maintaining sleep–wake cycles, energy metabolism, and neural network dynamics through the regulation of adenosine signaling, extracellular ion composition, and glycolytic pathways (62).

Neuroendocrine signaling, especially through the HPA axis, plays a crucial role in regulating glymphatic function. Elevated corticotropin-releasing hormone and cortisol levels, common in chronic stress and insomnia, impair AQP4 polarization and astrocytic coupling, thereby reducing cerebrospinal fluid-mediated clearance efficiency. Prolonged HPA axis activation disrupts cerebral blood flow and extracellular ion balance, compounding glymphatic dysfunction. Conversely, reduced glymphatic clearance leads to accumulation of neurotoxic metabolites, further stimulating HPA axis activity and establishing a vicious cycle between neuroendocrine dysregulation and impaired sleep homeostasis.

Additionally, MGBA-mediated gut dysbiosis influences this process by increasing BBB permeability and enabling microbial metabolites such as SCFAs and LPS to access the CNS. These metabolites modulate astrocyte and microglial activity, alter inflammatory tone, and further impact glymphatic efficiency. Consequently, the convergence of neuroendocrine signaling, GM dysregulation, and impaired glymphatic clearance promotes sleep fragmentation and contributes to neuropsychiatric comorbidities, including depression, anxiety, and cognitive decline (63, 64).

### 3.5 Therapeutic strategies targeting the microbiota-gut-brain axis

Given that the MGBA axis encompasses complex, multi-level interactions involving GM composition, microbial metabolite signaling, neuroendocrine pathways, immune-inflammatory responses, intestinal barrier integrity, and circadian rhythm oscillations, its inherent complexity poses significant challenges for conventional single-target therapeutic strategies in effectively addressing network dysregulation. Such approaches often yield limited efficacy or are associated with various adverse effects. Consequently, multi-target and multi-level intervention strategies based on holistic network regulation of the MGBA have emerged as a critical focus of current research and future clinical translation. In addition, this review categorizes these emerging MGBA-targeted interventions into eight principal classes, summarized in Table 1, based on their primary mechanisms of action to provide a systematic overview of their respective targets.

**Table 1:** Targeted therapeutic strategies for sleep disorders via the gut-brain axis.

Intervention	Clinical/basic research	Disease/model	Mechanism of action	Author and year of publication
Microbiota-targeted interventions				
Probiotics	Clinical Research	MDD patients with sleep disorder	• Increased abundance of *A. muciniphila* • Vagal nerve activation • Improved sleep and emotional regulation via gut-brain signaling	(87)
Probiotics	Clinical Research	Stress-induced insomnia	• Regulation of HPA axis activity (cortisol suppression) • Increased serum daidzein level	(88)
Probiotics	Basic research	PCPA-induced mice	• *A. muciniphila* enrichment • Inhibition of microglial overactivation and synaptic engulfment • Protection of hippocampal synaptic integrity and cognitive function	(89)
Probiotics	Basic research	CSR (chronic sleep restriction)	• Reduced peripheral & brain inflammation • Restored gut-brain axis hormones (ghrelin, leptin, GLP-1) • Enhanced antioxidant capacity	(90)
FMT	Clinical Research	PACS patients with insomnia	• *Gemmiger formicilis* enrichment • Menaquinone pathway suppression • Cortisol reduction	(91)
Probiotic supplementation	Basic research	Sleep deprivation	• Increased adenosine production • A2A receptor-mediated microglial activation • Oxidative stress and dopaminergic neuron loss • ADO-A2AR signalling-microglial activation-oxidative stress cascade	(92)
*A. muciniphila* supplementation	Basic research	Sleep deprivation	• Elevation of acetate and butyrate from *A. muciniphila* • Preservation of hippocampal synapses • Suppression of microglial synaptic engulfment	(93)
Herbal and multi-component pharmacological modulation				
Probiotic-fermented Gastrodia elata Blume	Basic research	Pentylenetetrazole-induced insomnia	• Modulation of gut microbiota composition • Regulation of neuroactive ligand-receptor interactions and actin cytoskeleton pathways • Anti-apoptotic/neuroprotective effects in insomnia model	(94)
Wendan Decoction	Basic research	PCPA-induced insomnia rats	• Inhibition of IDO1/KMO (kynurenine pathway) • Restoration of intestinal barrier via Occludin, Claudin-1, and ZO-1 upregulation • Gut microbiota modulation	(95)
Liuwei Anshen Capsule	Basic research	Sleep deprivation	• MAPK pathway inhibition • Regulation of apoptosis proteins (caspase-3, Bax, Bcl-2) • Suppression of neuroinflammation (TNF-α, IL-1β, IL-6)	(96)
Shuangxia Decoction	Basic research	Sleep deprivation	• Nrf2 pathway activation • Intestinal ROS suppression and barrier enhancement • Gut microbiota regulation • Hypothalamic purine metabolism modulation	(97)
Niacinamide	Basic research	Sleep deprivation	• Restoration of niacinamide-NAD^+^ metabolic pathway • Improvement of oocyte mitochondrial/meiotic competence • Amelioration of POI-related hormone/protein alterations	(98)
Neural and neurochemical circuit regulation				
TaVNS	Clinical Research	Primary insomnia	• Modulation of basal forebrain-cortical connectivity (visual, sensorimotor, mPFC) • Reduction in BF-cortex hyperconnectivity associated with insomnia severity	(99)
RE neuron activation	Basic research	Sleep deprivation	• Activation of thalamic nucleus reuniens neurons • CaMKII-dependent synaptic strengthening in RE-ZI pathway • Encoding of sleep need and promotion of deep NREM recovery sleep	(100)
Modulation of neuronal H₂O₂ levels	Basic research	Sleep deprivation	• Activation of TRPM2 by elevated H₂O₂ in sleep neurons • Induction of sleep initiation by moderate H₂O₂ • Excessive H₂O₂ triggers neuroinflammation and sleep fragmentation	(101)
Metabolic and immune-inflammatory pathway modulation				
Acetate supplementation	Basic research	Chronic sleep fragmentation	• Activation of pyruvate carboxylase in hypothalamic astrocytes • Restoration of glycolysis and TCA cycle flux	(102)
Choline supplementation	Basic research	Sleep deprivation	• Suppression of neuroinflammation and oxidative stress • Remodeling of hippocampal phospholipid profile • Preservation of hippocampal integrity and BBB function	(103)
Marine n-3 PUFA supplementation	Clinical Research	Type 2 diabetes mellitus with sleep disorders	• RORα targeting and BMAL1 nuclear translocation. • Activation of core clock genes (CLOCK / BMAL1 / PER2). • Circadian entrainment and suppression of HPA overactivity	(104)
Chronobiological and circadian rhythm regulation				
Melatonin treatment	Basic research	Sleep deprivation	• Reduced Aeromonas, increased Lachnospiraceae_NK4A136 and butyrate • TLR4/NF-κB pathway inhibition • Attenuated hippocampal neuroinflammation	(105)

## 4 Spectulation and limits

Accumulating evidence suggests the pivotal involvement of the MGBA in the pathogenesis of sleep disorders, providing a solid mechanistic basis for targeted therapeutic exploration. Collectively, this body of work suggests a bidirectional pathological cycle between the MGBA and sleep regulation: gut dysbiosis impairs microbial metabolite signaling and promotes neuroinflammation, thereby disrupting sleep, whereas sleep deprivation further aggravates dysbiosis by altering gut motility, permeability, and immune balance (65, 66). Microbial-derived metabolites, such as tryptophan derivatives and phenethylamine, can promote melatonin synthesis and influence sleep–wake regulation, underscoring the potential of microbiota-targeted therapeutic approaches (67). Conversely, dysbiosis-induced activation of the NLRP3 inflammasome, a central component of the innate immune system, links intestinal imbalance to systemic and neuroinflammatory cascades that contribute to cognitive impairment and neurodegenerative diseases, including Alzheimer’s disease (68). Importantly, Mendelian randomization analyses have provided evidence supporting a bidirectional causal relationship between MGBA dysfunction and sleep disorders, reinforcing that microbial function and host pathophysiology are profoundly context dependent. This reciprocal interplay constitutes a fundamental mechanism underlying both the persistence and therapeutic refractoriness of sleep disturbances (66).

In recent years, the integration of multi-omics technologies, such as metagenomics, metabolomics, and transcriptomics, has significantly deepened our understanding of the MGBA and its role in sleep regulation. These approaches have identified specific microbial taxa such as Lactobacillus, Bifidobacterium, and *Akkermansia muciniphila* that influence tryptophan metabolism and melatonin synthesis, thereby providing a mechanistic explanation for their sleep-promoting effects. Moreover, metabolomic profiling in sleep-deprived models has revealed significant reductions in SCFAs, indole derivatives, and secondary bile acids—key mediators of gut-brain signaling. These findings underscore the power of systems biology approaches in delineating microbial-neurochemical interactions underlying sleep–wake homeostasis.

**Figure 2:**
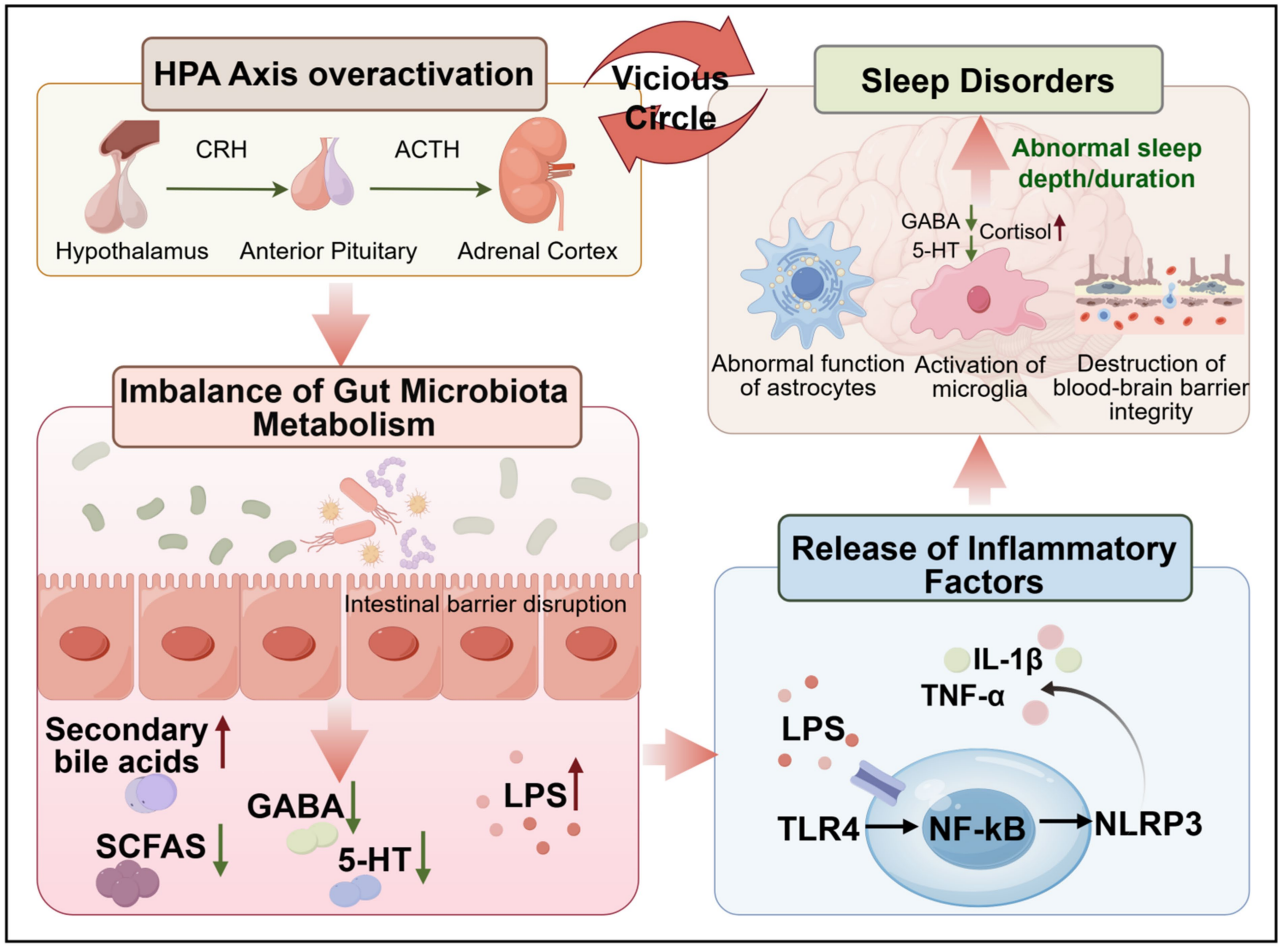
Hypothalamic secretion of CRH stimulates the release of ACTH from the adenohypophysis, which in turn promotes the secretion of cortisol from the adrenal cortex, resulting in the excessive activation of the HPA axis. Abnormal activation of the HPA axis not only directly leads to abnormal sleep depth and sleep time, but also disrupts the balance of intestinal flora (such as secondary bile acids, SCFAs, GABA and 5-HT abnormalities) and the integrity of the intestinal barrier, allowing LPS to enter the blood. LPS induces the activation of the NLRP3 inflammasome by activating the TLR4/NF-κB pathway, releasing proinflammatory factors such as IL-1β and TNF-*α*. These factors aggravate the damage of the intestinal barrier, destroy the blood–brain barrier, activate microglia in the brain, interfere with the function of astrocytes, and eventually form a vicious circle of “intestinal flora disorder, inflammatory response, HPA axis over-activation, sleep disorders,” and aggravate sleep pathological state.

Within this framework, microbial metabolites function not only as mediators of gut-brain communication but also as initiators of neuroimmune cascades. Specifically, TLR4/NF-κB, ATP/P2X7R, and NLRP3/caspase-1 signaling axes form a self-sustaining inflammatory loop that amplifies neural dysfunction. LPS from dysbiotic Gram-negative bacteria prime the TLR4/NF-κB pathway, extracellular ATP released during sleep disruption activates P2X7R, and subsequent NLRP3-driven IL-1β secretion amplifies neuroinflammation and suppresses GABAergic transmission—hallmarks of insomnia and hyperarousal (69–71). Activated microglia further perpetuate this process by downregulating GABA synthesis and inhibitory tone, creating a persistent neuroinflammatory state that reinforces sleep fragmentation (72). Thus, the TLR4-P2X7R-NLRP3 axis represents a central pathological cascade linking gut-derived signals to neural excitability. Targeting this cascade may provide a mechanistic basis for next-generation therapies aimed at restoring sleep homeostasis and mitigating related neuropsychiatric comorbidities (Figure 2).

Building upon these mechanisms, emerging therapeutic strategies targeting the MGBA for sleep disorders demonstrate promising potential. The core strategy employs comprehensive, multi-pronged interventions such as microbiota-targeted interventions, herbal and multi-component pharmacological modulation, neural and neurochemical circuit regulation, metabolic and immune-inflammatory pathway modulation and chronobiological and circadian rhythm regulation, to achieve precise manipulation of the gut ecosystem. Emerging evidence strongly supports the efficacy of such strategies. Parallel to these developments, the field is moving toward personalized and precision-based microbiota therapeutics. Interindividual variability in microbial composition and metabolite profiles profoundly shapes responses to probiotics and fecal microbiota transplantation (FMT). Machine learning and artificial intelligence algorithms are increasingly being utilized to identify predictive microbial signatures associated with sleep quality and therapeutic responsiveness. Integrating clinical metadata, metagenomic datasets, and behavioral phenotypes through AI modeling may soon enable individualized microbial interventions that optimize treatment efficacy while minimizing adverse outcomes. Moreover, interventions that strengthen intestinal barrier integrity may reduce LPS translocation and subsequent systemic inflammation, thereby attenuating HPA axis overactivation and cortisol-mediated sleep disruption. Beyond traditional probiotics and FMT, next-generation microbiota-based therapeutics are emerging as promising modalities. Engineered bacterial consortia capable of synthesizing neurotransmitters (GABA, 5-HT) or anti-inflammatory metabolites (butyrate) have been developed to precisely modulate host neurochemistry (73). Synthetic biology and CRISPR-based technologies now enable the design of “designer microbes” that secrete targeted molecules directly within the intestinal lumen. In addition, dietary interventions rich in prebiotic fibers, polyphenols, and omega-3 fatty acids demonstrate synergistic effects with probiotic supplementation, suggesting that nutritional modulation of the MGBA may serve as a safe, noninvasive adjunct to pharmacotherapy. It is noteworthy that the development of future MGBA-targeted therapies should incorporate personalized and precision-based approaches, reflecting the considerable interindividual variation in GM composition shaped by genetic, environmental, dietary, and other factors (74).

Although the present review provides a comprehensive overview of the role of the MGBA in sleep disorders, several important limitations and controversies remain in this field. First, the causal relationship between GM alterations and sleep disturbances remains to be clarified. Most available evidence is derived from cross-sectional studies, which can only reveal correlations but not causality. Mendelian randomization analyses offer an alternative approach for causal inference, yet their conclusions are often constrained by the strength of genetic instruments, population specificity, and assumptions of linearity, thereby limiting the generalizability of findings (75). Second, the lack of repetition and methodological standardization in microbiome-based interventions represents a major obstacle to clinical translation. Despite growing enthusiasm for probiotic and FMT therapies, significant heterogeneity in strain selection, dosage, formulation, and delivery routes contributes to inconsistent outcomes across trials (76, 77). Even closely related Lactobacillus or Bifidobacterium strains can produce distinct molecular metabolites and elicit divergent host responses, underscoring the importance of strain-level precision (77). Variations in probiotic viability, administration duration, and host colonization capacity further complicate reproducibility (78). Similarly, the absence of standardized FMT protocols—including donor screening, microbial processing, concentration, and administration methods—results in substantial variability in efficacy and safety, hindering cross-study comparability and large-scale meta-analyses (76). Third, host-specific factors profoundly influence therapeutic responses. Genetic background, baseline microbiome composition, diet, sleep–wake rhythm, medication use, and environmental exposures all modulate the efficacy of MGBA-targeted interventions (79). For instance, individuals with distinct microbial enterotypes or metabolic capacities may respond differently to the same probiotic strain or FMT donor, suggesting that host-microbe compatibility is a key determinant of therapeutic success. Failure to account for these factors may explain much of the inconsistency observed across clinical trials (80). Fourth, current mechanistic studies heavily rely on animal models such as germ-free or sleep-deprived rodents. While these models are invaluable for hypothesis generation, substantial interspecies differences in physiology, sleep architecture, metabolism, and microbiome ecology limit the extrapolation of preclinical findings to humans (81). Moreover, many studies focus on single microbial metabolites or inflammatory biomarkers (IL-6, TNF-*α*, cortisol), without capturing the dynamic interplay among neurotransmitters (5-HT, dopamine, GABA), immune mediators, and endocrine pathways that jointly influence sleep regulation (82). Finally, clinical translation remains limited by methodological constraints, including small sample sizes, short follow-up periods, and inconsistent outcome evaluations, which collectively impede the reliable assessment of long-term efficacy and safety (83).

To overcome these challenges and unlock the therapeutic potential of the MGBA in sleep medicine, future research should focus on: (1) identifying functionally defined microbial strains and clarifying their mechanistic pathways; (2) developing standardized, reproducible protocols for probiotic and FMT interventions, with transparent reporting of strain taxonomy, viable CFU counts, formulation, donor screening, and administration routes; (3) integrating multi-omics and host phenotyping to capture host–microbe interactions and guide precision-microbiome therapeutics; (4) adopting large-scale, prospective, and mechanistically informed trials to establish causal and temporal relationships between microbiota dynamics and sleep disorders (84). Additionally, future investigations should explore circadian rhythm-microbiome interactions and sex-specific differences. Gut microbial activity displays diurnal rhythms that are synchronized with host circadian gene expression, and disruption of this alignment caused by shift work, jet lag, or chronic insomnia can lead to systemic metabolic disturbances and neuroinflammatory responses. Integrating chronobiological approaches into MGBA research could therefore reveal timing-based intervention strategies, while elucidating sex-related differences may provide insight into the higher prevalence of insomnia among females.

Given the multifactorial nature of sleep pathophysiology and the complexity of the MGBA, multimodal and personalized therapeutic strategies that integrate engineered microbial strains with nanotechnology-based delivery systems represent promising directions for future research (85). For example, nanomaterials can be designed to release therapeutic agents in response to specific microenvironmental cues, enabling spatiotemporal precision in microbial modulation (86). Such approaches may enhance bioavailability, improve targeting accuracy, and ultimately yield superior clinical efficacy.
